# Comment on “Physical Exercise and Patients with Chronic Renal Failure: A Meta-Analysis”

**DOI:** 10.1155/2018/3826159

**Published:** 2018-06-05

**Authors:** Mohammad Alwardat

**Affiliations:** School of Neuroscience, Faculty of Medicine and Surgery, University of Rome “Tor Vergata”, Rome, Italy

Qiu et al [1] recently conducted a systematic review and meta-analysis that evaluated the effects of exercise on the health of patients with chronic renal failure. Investigation of the effect of physical activity and exercise can enhance the awareness of these nonpharmacological interventions on physical and mental health for patients with chronic renal failure. However, we have some comments with respect to the procedures and results of this study.

First, in the final paragraph of the introduction, the authors mention that “several published randomized controlled trials (RCT) studies about the effect of exercise on patients with renal failure have shown inconsistent results [12, 14–16].” This statement is referenced with 4 studies (Geary et al., 1990; Jungers et al., 2000; Adams and Vaziri 2006; and Salako et al., 2002), all of which in fact are not RCTs. Moreover, Geary, et al. (1990) expressed their opinion in a short communication about the role of nutrition in neurologic health and development of infants with chronic renal failure. In addition, Jungers et al. (2000) conducted a review to discuss the atherosclerotic complications in chronic renal failure, and Adams and Vaziri (2006) investigated in their review the effect of exercise on skeletal muscle dysfunction in chronic renal failure, and they concluded that regular exercise regimens can improve physical condition, biochemical profile, and, perhaps, mental performance in end-stage renal disease patients. Also, Salako et al. (2002) study the prevalence of hepatitis B and C viruses in predialysis patients with chronic renal failure, although the study did not mention any effect of exercise on patients with renal failure.

Second, the inclusion criteria are ambiguous. The authors do not sufficiently follow the PICO format (P: participants, I: intervention, C: comparison, O: outcomes). The authors state the inclusion criteria as follows: “studies must be conducted on adults; studies investigated the correlation between exercise and renal failure; the population in researches should be in dialysis; and full text of RCT is available.” Additionally, the authors state the review aims as follows: “evaluate the effects of exercise on the health of patients with chronic renal failure.” Surprisingly, the review did not clearly report the other components of inclusion criteria like comparison and outcome measures. The included articles were RCTs that compared the effect of different types of exercise on physical function and cardiorespiratory fitness in patients with chronic renal failure. Running a systematic review without full knowledge about the inclusion criteria can lead to problems with assessing the validity, applicability, and comprehensiveness of the systematic review [2].

Third, the systematic review is different from other types of literature reviews. It must provide an explicit, reproducible methodology and include a systematic search that attempts to identify all studies that would meet the eligibility criteria [3]. This unique construction requires the Methods section of a systematic review to be evaluated much like a quantitative research study. However, this review has also several troubling flaws in the methods. The authors reported using PubMed; there was also the opportunity to use Medical Subject Headings (MeSH) in the search. Using subject headings in addition to keywords is a key point of searching for studies according to Cochrane Handbook for Systematic Reviews of Interventions [3].

Finally, the authors include study with different intensities (Cho and Sohng, 2014), such as virtual reality exercise, a light-intensity activity. Including different intensities means that the review and meta-analysis include heterogeneous types of intervention, which makes firm conclusion about the impact, optimum type, and intensity of the exercise interventions be very difficult.
